# Left ventricular-arterial coupling is associated with prolonged mechanical ventilation in severe post-cardiac surgery patients: an observational study

**DOI:** 10.1186/s12871-018-0649-7

**Published:** 2018-12-06

**Authors:** Xu Wang, Yun Long, Huaiwu He, Guangliang Shan, Rui Zhang, Na Cui, Hao Wang, Xiang Zhou, Xi Rui, Wanglin Liu

**Affiliations:** 1 Department of Critical Care Medicine, Peking Union Medical College Hospital, Peking Union Medical College, Chinese Academy of Medical Science, 1 Shuaifuyuan, Dongcheng District, Beijing, 100730 China; 2 Department of Epidemiology and Biostatistics, Institute of Basic Medicine Sciences, Chinese Academy of Medical Sciences (CAMS) & School of Basic Medicine, Peking Union Medical College, Beijing, China

**Keywords:** Ventricular-arterial coupling, Severe cardiogenic shock, Post-cardiac surgery, Prolonged mechanical ventilation, Cardiac work efficiency cardiac reserve

## Abstract

**Background:**

Weaning post-cardiac surgery patients from mechanical ventilation (MV) poses a big challenge to these patients. Optimized left ventricular-arterial coupling (VAC) may be crucial for reducing the MV duration of these patients. However, there is no research exploring the relationship between VAC and the duration of MV. We performed this study to investigate the relationship between left ventricular-arterial coupling (VAC) and prolonged mechanical ventilation (MV) in severe post-cardiac surgery patients.

**Methods:**

This was a single-center retrospective study of 56 severe post-cardiac surgery patients from January 2015 to December 2017 at the Department of Critical Care Medicine of Peking Union Medical College Hospital. Patients were divided into two groups according to the duration of MV (PMV group: prolonged mechanical ventilation group, MV > 6 days; Non-PMV group: non-prolonged mechanical ventilation group, MV ≤ 6 days). Hemodynamics and tissue perfusion data were collected or calculated at admission (T0) and 48 h after admission (T1) to the ICU.

**Results:**

In terms of hemodynamic and tissue perfusion data, there were no differences between the two groups at admission (T0). Compared with the non-prolonged MV group after 48 h in the ICU (T1), the prolonged MV group had significantly higher values for heart rate (108 ± 13 vs 97 ± 12, *P* = 0.018), lactate (2.42 ± 1.24 vs.1.46 ± 0.58, *P* < 0.001), and Ea/Ees (5.93 ± 1.81 vs. 4.05 ± 1.20, *P* < 0.001). Increased Ea/Ees (odds ratio, 7.305; 95% CI, 1.181–45.168; *P* = 0.032) and lactate at T1 (odds ratio, 17.796; 95% CI, 1.377–229.988; *P* = 0.027) were independently associated with prolonged MV. There was a significant relationship between Ea/Ees_T1_ and the duration of MV (*r* = 0.512, *P* < 0.01). The area under the receiver operating characteristic (AUC) of the left VAC for predicting prolonged MV was 0.801, and the cutoff value for Ea/Ees was 5.12, with 65.0% sensitivity and 90.0% specificity.

**Conclusions:**

Left ventricular-arterial coupling was associated with prolonged mechanical ventilation in severe post-cardiac surgery patients. The assessment and optimization of left VAC might be helpful in reducing duration of MV in these patients.

## Background

Prolonged mechanical ventilation (PMV) is required by 2.4–9.9% of post-cardiac surgery patients and is associated with increased medical resource use and mortality [1–4]. Cardiac function has been identified as an important factor during weaning from mechanical ventilation (MV). MV has several advantages effects on hemodynamics in post-cardiac surgery patients with poor cardiac function, including reducing venous return with low cardiac preload and decreasing the afterload of the left ventricle (LV) [5]. In contrast, weaning from MV with increased left ventricular preload and afterload may pose a considerable challenge for these patients [5, 6].

Several investigators have found that low cardiac output (CO) was associated with PMV in post-cardiac surgery patients [7, 8]. However, in some research, patients with normal CO value might also be difficult to wean [9]. Some more superior indicators need to be explored to assess the possibility of PMV.

The LV pumps blood to the arterial vessel, which is the output of energy [10]. Importantly, weaning from a mechanical ventilator challenges the heart, and the heart should generate more stroke work (SW) in response to the challenge. Cardiac work efficiency is the ratio of SW to total mechanical energy [11]. The higher efficiency is, the more SW a heart can produce with a given total energy. Therefore, cardiac work efficiency might play an important role in the weaning process.

**Fig. 1:**
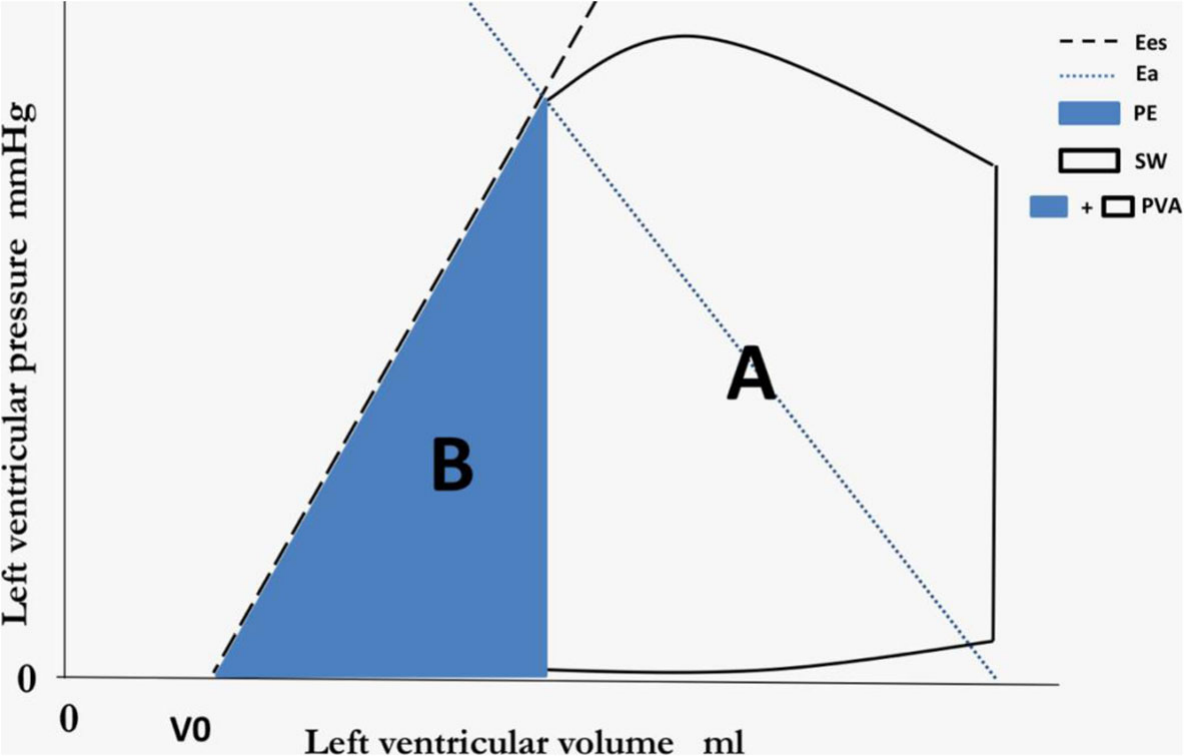
Analysis of ventricular-arterial coupling in the pressure-volume plane. A pressure-volume loop is shown along with the end-systolic pressure-volume relationship (black dashed line). The unfilled “A” area represents stroke work; the blue “B” area represents potential energy. EES is the slope of the end-systolic pressure-volume relationship. EA (blue dashed line) is plotted on top of the pressure-volume loop. V0 is the volume-axis intercept of the end-systolic pressure- volume relationship, representing the LV volume at zero intracavitary pressure. The pressure-volume area (PVA) is the sum of potential energy and stroke (i.e., external) work. The PVA represents the total mechanical energy generated by LV contraction until the end of systole

As we all know, left ventricular-arterial coupling (VAC) was used to assess cardiac work efficiency [12]. There are two parameters in VAC: Ea and Ees. Ea is the arterial end-systolic elastance, the slope of blue dotted line, which reflects afterload, and Ees is the left ventricular end-systolic elastance, the slope of black dotted line, which represents contractility (Fig. 1). VAC, the ratio of Ea to Ees, can appropriately reflect the proportion between area A and area B (Fig. 1).

For a given beat, the pressure–volume area (PVA, SW plus potential energy, Fig. 1) stands for the total mechanical energy and correlates strongly with myocardial oxygen consumption (MVO2) [13, 14]. The ratio of SW to PVA, which can be estimated by proportion of area B to area A, is representative of cardiac work efficiency. As we have discussed above, VAC can reflect the proportion of area A to area B, so it is understandable that VAC can be a surrogate for cardiac work efficiency [12, 15]. A heart with a high Ea/Ees usually presents an increased proportion of the PVA corresponding to potential energy rather than stroke work, denoting an unfavorable energy efficiency state [11]. In contrast, a more efficient heart with a lower Ea/Ees can consume less energy to attain suitable SW and thus has a greater SW reserve. Thus, VAC can reflect efficiency as well as SW reserve. When the VAC is appropriate, the heart have more power potential to face the challenge of weaning. Here, we hypothesized that left VAC is associated with the duration of MV.

To the best of our knowledge, the predictive value of VAC for PMV has not been investigated in severe post-cardiac surgery patients. Therefore, we performed this study to investigate whether left VAC can predict PMV in this patient population.

## Methods

### Patients

We performed a retrospective single-center study of severe cardiac patients who were admitted to the ICU after cardiac surgery with continuous PiCCO monitors (Pulsio Cath PV 2015 L20: Pulsion Medical Systems: Munich, Germany) for unstable hemodynamics from January 2015 to December 2017 at the Department of Critical Care Medicine of Peking Union Medical College Hospital. These patients had a preoperative class III or IV New York Heart Association (NYHA) classification. The exclusion criteria include 1. Discharged within 48 h; 2. Discharged against medical advice or died in the first 6 days; 3. Acquired pneumonia or serious cerebrovascular accidents in the first 6 days of ICU admission.

### Data collection

A standardized weaning protocol has been applied for all included patients. We divided patients into two groups according to the duration of MV (PMV group: prolonged MV group, defined as MV > 6 days; non-PMV group: non-prolonged MV group, defined as MV ≤6 days). Medical records were reviewed to obtain information about gender, age, concomitant diseases, types of surgery, the 1st day SOFA, the duration of extracorporeal circulation (ECC), vasopressor and inotrope treatment, and the duration of MV and ICU stay. We also collected hemodynamics and tissue perfusion data at two time points (T0: admission; T1: 48 h after admission; both time points were approximate, within +/− 3 h) from the Critical Care Monitor System of the Department of Critical Care Medicine at Peking Union Medical College Hospital, which recorded real-time clinical data from bedside equipment. This system is maintained by Donghua software cooperation through DtHealth system. We also collected the PaO2/FiO2 (P/F) data on the first and second day in the ICU.

### Study definitions

- Severe post-cardiac surgery patients defined as after conventional treatments, patients still have the following problems: a. lactate clearance less than 30% in the first 24 h;b. high dose of norepinephrine, more than 1μg/kg/min; c. undefined shock type, requiring more accurate monitoring techniques, like PiCCO.
- PMV group: prolonged MV group, defined as MV > 6 days; non-PMV group: non-prolonged MV group, defined as MV ≤6 days.
- End-systolic aortic pressure can be estimated as 0.9 times the peak brachial systolic pressure. In this study, we calculated Ea as 0.9*SBP divided by SV (stroke volume), which can be expressed as Ea = 0.9 SBP/SV.
- The simplified formula for calculating Ees was Ees = SBP/(GEDV/4-SV). Technically, Ees = ESP/(ESV-V0). ESP is LV end-systolic pressure and ESV is LV end-systolic volume. V0 represents a purely theoretical LV volume at zero intracavitary pressure [16]. We considered ESP as 0.9*SBP [16] and left out V0. Global end-diastolic volume (GEDV) divided by 4 was used as the left ventricular end-diastolic volume.

### Statistical analysis

A descriptive analysis was performed. Continuous variables are expressed as the mean ± standard deviation, and categorical variables are expressed as absolute values and percentages. For continuous variables, the data were analyzed using Student’s t-test, the Mann-Whitney U test or the Kruskal-Wallis test depending on data distribution and the number of variables. Categorical variables were analyzed using the chi-square test or Fisher’s test.

Variables were introduced into a multivariable binary logistic regression model if they were significantly associated with prolonged MV in the univariate analysis (*p* value < 0.2). General demographics were also used in the model. Discrimination of values was performed using the receiver operating characteristic (ROC) analysis. All comparisons were two-tailed, and *P* < 0.05 was required to exclude the null hypothesis. The statistical analysis was performed using IBM SPSS Statistics, Version 20.0 (Armonk, NY: IBM Corp).

## Results

**Fig. 2:**
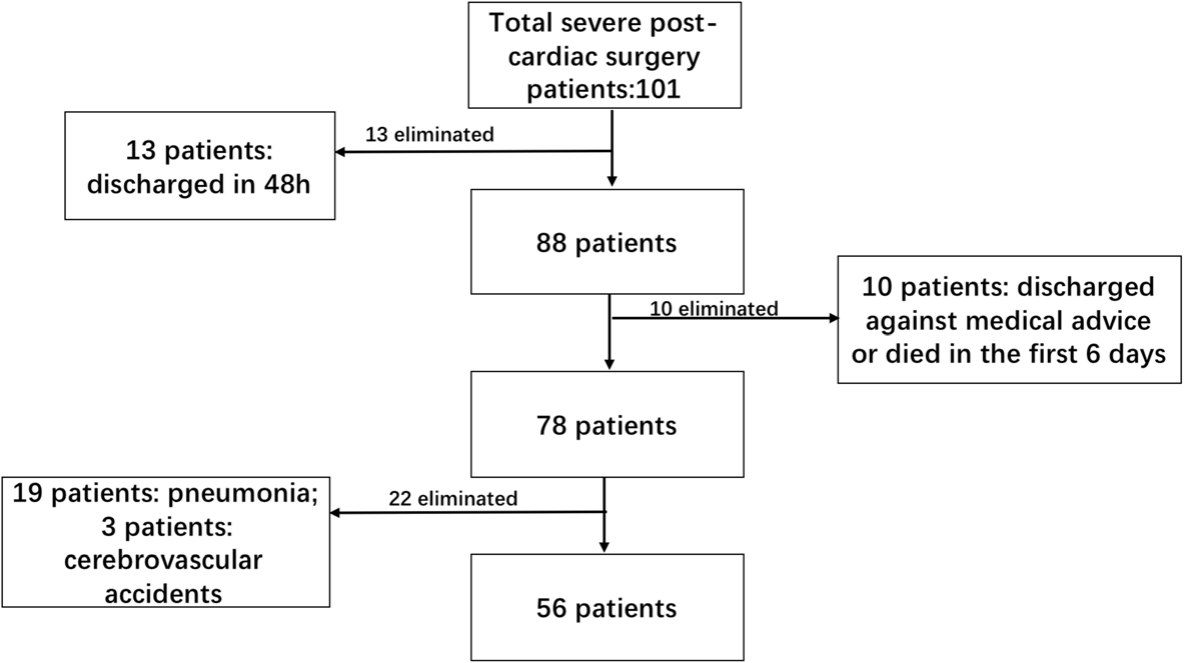
Flow diagram showing the enrollment of patients

During the study period, a total of 101 severe post-cardiac surgery patients were admitted to our department, and 45 were excluded for various reasons: 13 were discharged within 48 h; 10 were discharged against medical advice or died in the first 6 days; and 19 acquired pneumonia and 3 had serious cerebrovascular accidents in the first 6 days after cardiac surgery in the ICU. Therefore, 56 patients were enrolled in this study. The flow diagram for patient enrollment is shown in Fig. 2. There were 26 patients in the PMV group and 30 patients in the non-PMV group.

### Demographics and clinical characteristics

There were no significant differences in gender, age, types of surgery (CABG (coronary artery bypass grafting), pericardiectomy, valve surgery, ventricular septal defect repair, atrial neoplasm resection, aorta replacement) or concomitant diseases (hypertension, diabetes, CAC, CKD, CHF) between the two groups. The PMV group had a significantly higher SOFA score than the non-PMV group (PMV group vs non-PMV group: 12.56 ± 2.71 vs 11.09 ± 2.01, *P* = 0.003). No significant differences were found in the use of inotropes between the two groups during the three periods. The demographics and clinical characteristics of all the patients are presented in Table 1.

**Table 1:** The demographics and clinical characteristics of all the patients

Characteristics	Groups		*P*
	Non-prolonged MV (*n* = 30)	Prolonged MV (*n* = 26)	
Gender Males *n* (%)	23 (76.67%)	22 (84.62%)	0.455
Age (years)	49.90 ± 16.60	54.27 ± 16.52	0.329
SOFA	11.09 ± 2.01	12.56 ± 2.71	0.003
MV (hours)	97.33 ± 26.97	280.50 ± 141.65	< 0.001
ICU LOS (days)	8.13 ± 4.14	15.73 ± 7.02	<0.001
Types of surgery			
CABG n (%)	6 (20.00%)	5 (19.23%)	0.942
Pericardiectomy *n* (%)	10 (33.33%)	13 (50.00%)	0.206
Valve surgery *n* (%)	13 (43.33%)	9 (34.62%)	0.505
Ventricular septal defect repair *n* (%)	0 (00.00%)	1 (3.85%)	0.464
Atrial neoplasms resection *n* (%)	2 (6.67%)	0 (0.00%)	0.282
Replacement of aorta *n* (%)	1 (3.33%)	0 (0.00%)	1.000
ECC duration (min)	116.21 ± 98.56	82.56 ± 79.50	0.200
Concomitant diseases			
Hypertension *n* (%)	11 (36.67%)	6 (23.08%)	0.270
Diabetes n (%)	7 (23.33%)	3 (11.54%)	0.310
CAC *n* (%)	8 (26.67%)	5 (19.23%)	0.511
CKD *n* (%)	4 (13.33%)	1 (3.85%)	0.358
CHF *n* (%)	8 (26.67%)	9 (34.62%)	0.519
Post-surgery drugs			
Vasopressors			
0-24 h *n* (%)	28 (93.33%)	26 (100%)	0.494
24-48 h *n* (%)	25 (83.33%)	26 (100%)	0.055
> 48 h n (%)	24 (80.00%)	26 (100%)	< 0.001
Intropes			
0-24 h *n* (%)	28 (93.33%)	25 (96.15%)	1.000
24-48 h *n* (%)	28 (93.33%)	25 (96.15%)	1.000
> 48 h *n* (%)	19 (63.33%)	20 (76.92%)	0.384

*MV* mechanical ventilation, *SOFA* sequential organ failure assessment, *LOS* length of stay, *CABG* coronary artery bypass grafting, *ECC* extracorporeal circulation, *CAC* coronary atherosclerotic cardiopathy, *CKD* chronic kidney disease, *CHF* chronic heart failure

### Relationship between hemodynamic parameters and the duration of MV

There were no differences in HR_T0_ (heart rate), SBP_T0_ (systolic blood pressure), CVP_T0_ (central venous pressure), P/F_T0_ (PaO_2_/FiO_2_), lactate_T0_, CI_T0_ (cardiac index), SVI_T0_ (stroke volume index), SVRI_T0_ (systemic vascular resistance index), Ea_,T0_, Ees_,T0_ or Ea/Ees_,T0_ between the two groups (Table 2). Compared to the non-PMV group, the PMV group had significantly higher values for HR_T1_ (*P* = 0.018), lactate_T1_ (*P* < 0.001), and Ea/Ees_,T1_ (4.05 ± 1.20 VS 5.93 ± 1.81, *P* < 0.001) and lower values for Ees_,T1_ (*P* = 0.022), SVI_T1_ (*P* = 0.049), SBP_T1_ (*P* = 0.014).

**Table 2:** Comparison of hemodynamic parameters and tissue perfusion between the two groups

Variables	Times	Non-prolonged MV	n	Prolonged MV	n	*P*
HR bpm	T0	103 ± 15	30	110 ± 17	26	0.113
	T1	97 ± 13	30	108 ± 13	26	0.018
SBP mmHg	T0	129 ± 17	30	129 ± 15	26	0.938
	T1	131 ± 17	30	118 ± 22	26	0.014
CVP mmHg	T0	10.03 ± 2.80	30	11.08 ± 3.72	26	0.237
	T1	10.59 ± 2.15	29	9.35 ± 2.55	26	0.055
P/F mmHg	T0	355.00 ± 172.43	30	291.65 ± 91.29	23	0.092
	T1	293.90 ± 93.65	29	280.48 ± 120.81	25	0.648
T °C	T0	36.03 ± 0.79	30	35.87 ± 0.66	26	0.406
	T1	36.78 ± 0.61	30	36.63 ± 0.72	26	0.319
Lac mmol/L	T0	5.56 ± 3.80	30	5.66 ± 3.99	23	0.928
	T1	1.46 ± 0.58	29	2.42 ± 1.24	25	< 0.001
CI L/min/m^2^	T0	2.89 ± 0.81	18	2.90 ± 0.87	17	0.988
	T1	2.82 ± 0.63	21	2.59 ± 0.80	21	0.318
SVI ml/m^2^	T0	27.12 ± 7.75	18	26.24 ± 8.04	17	0.887
	T1	29.52 ± 10.84	21	23.58 ± 6.75	21	0.049
SVRI dyn.sec.cm^−5^.m^2^	T0	2399.06 ± 1146.16	17	2255.71 ± 883.40	17	0.615
	T1	2260.71 ± 668.84	24	2309.76 ± 555.38	23	0.980
Ea mmHg/ml	T0	2.62 ± 1.07	18	2.86 ± 1.38	17	0.566
	T1	2.57 ± 1.22	21	2.93 ± 0.91	21	0.279
Ees mmHg/ml	T0	0.570 ± 0.175	18	0.523 ± 0.109	17	0.346
	T1	0.578 ± 0.179	21	0.461 ± 0.136	21	0.022
Ea/Ees	T0	4.39 ± 1.90	18	4.89 ± 1.80	17	0.423
	T1	4.05 ± 1.20	21	5.93 ± 1.81	21	< 0.001

*MV* mechanical ventilation, *HR* heart rate, *SBP* systolic blood pressure, *CVP* central venous pressure, *P/F* PaO2/FiO2, *T* temperature, *Lac* lactate, *CI* cardiac output index, *SVI* stroke volume index, *SVRI* Systemic vascular resistance index, *Ea* arterial end-systolic elastance, *Ees* left ventricular end-systolic elastance, *Ea/Ees* ventricular arterial coupling, *T0* at admission, *T1* 48 h after admission

**Fig. 3:**
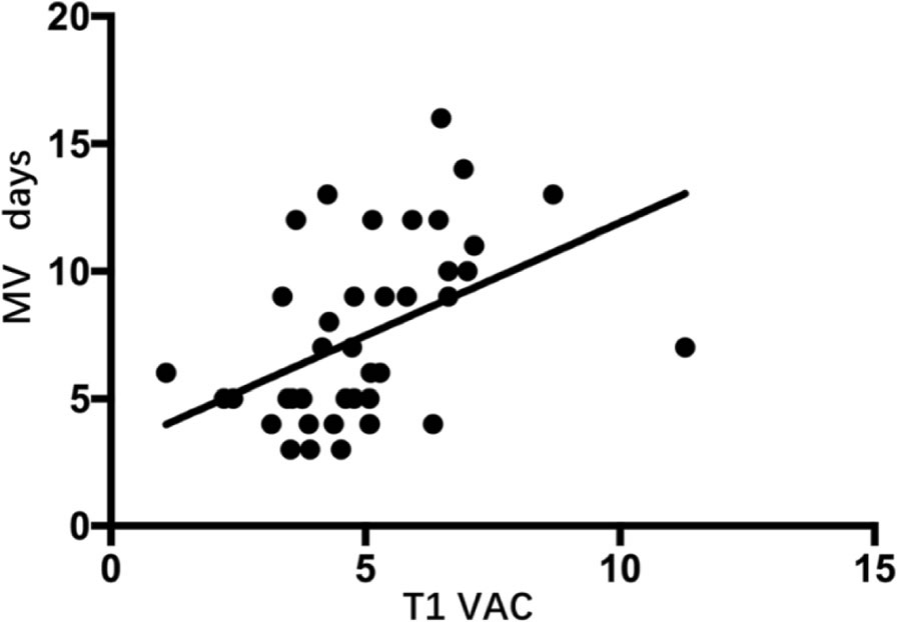
The relationship between Ea/Ees at T1 and the duration of MV. r^2^ = 0.262

There was a significant correlation between Ea/Ees,_T1_and the duration of MV (Fig. 3); the correlation coefficient was 0.512 (Ea/Ees,_T1,_*P* < 0.01). There were no correlations between SVI_T1_ or CI_T1_ and the duration of MV.

### Multivariable binary logistic regression

The multivariable logistic regression analysis of the data at T1 demonstrated that SBP_T1_ (OR 0.921; 95% CI 0.853–0.994, *P* < 0.05), Ea/Ees_,T1_ (OR 6.840; 95% CI 1.031–45.359, *P* < 0.05) and lactate_T1_ (OR 15.269; 95% CI 1.231–189.427, *P* < 0.05) were independent predictors of PMV in severe post-cardiac surgery patients (Table 3).

**Table 3:** Multivariate logistic regression analysis for possible risk factors of prolonged mechanical ventilation

Variable	B	SE	Wald	P	OR	95% CI for OR Lower Upper	
Multivariate							
Age years	0.040	0.050	0.625	0.429	1.040	0.943	1.148
SOFA	0.012	0.361	0.001	0.974	1.012	0.498	2.055
T1 SBP mmHg	−0.083	0.039	4.495	0.034	0.921	0.853	0.994
T1 HR bpm	0.103	0.085	1.459	0.227	1.108	0.938	1.310
T1 CVP mmHg	−0.300	0.278	1.164	0.281	0.741	0.430	1.277
T1 CI L/min/m^2^	−1.771	1.788	0.982	0.322	0.170	0.005	5.657
T1 SVI ml/m^2^	0.196	0.183	1.150	0.284	1.216	0.850	1.739
T1 Ea/Ees	1.923	0.966	3.965	0.046	6.840	1.031	45.395
T1 Lac mmol/L	2.726	1.285	4.501	0.034	15.269	1.231	189.427

*HR* heart rate, *SBP* systolic blood pressure, *CVP* central venous pressure, *CI* cardiac output index, *SVI* stroke volume index, *Ea/Ees* ventricular arterial coupling, *Lac* lactate, *T0* at admission, *T1* 48 h after admission

### Predictive value of three variables in prolonged mechanical ventilation

**Fig. 4:**
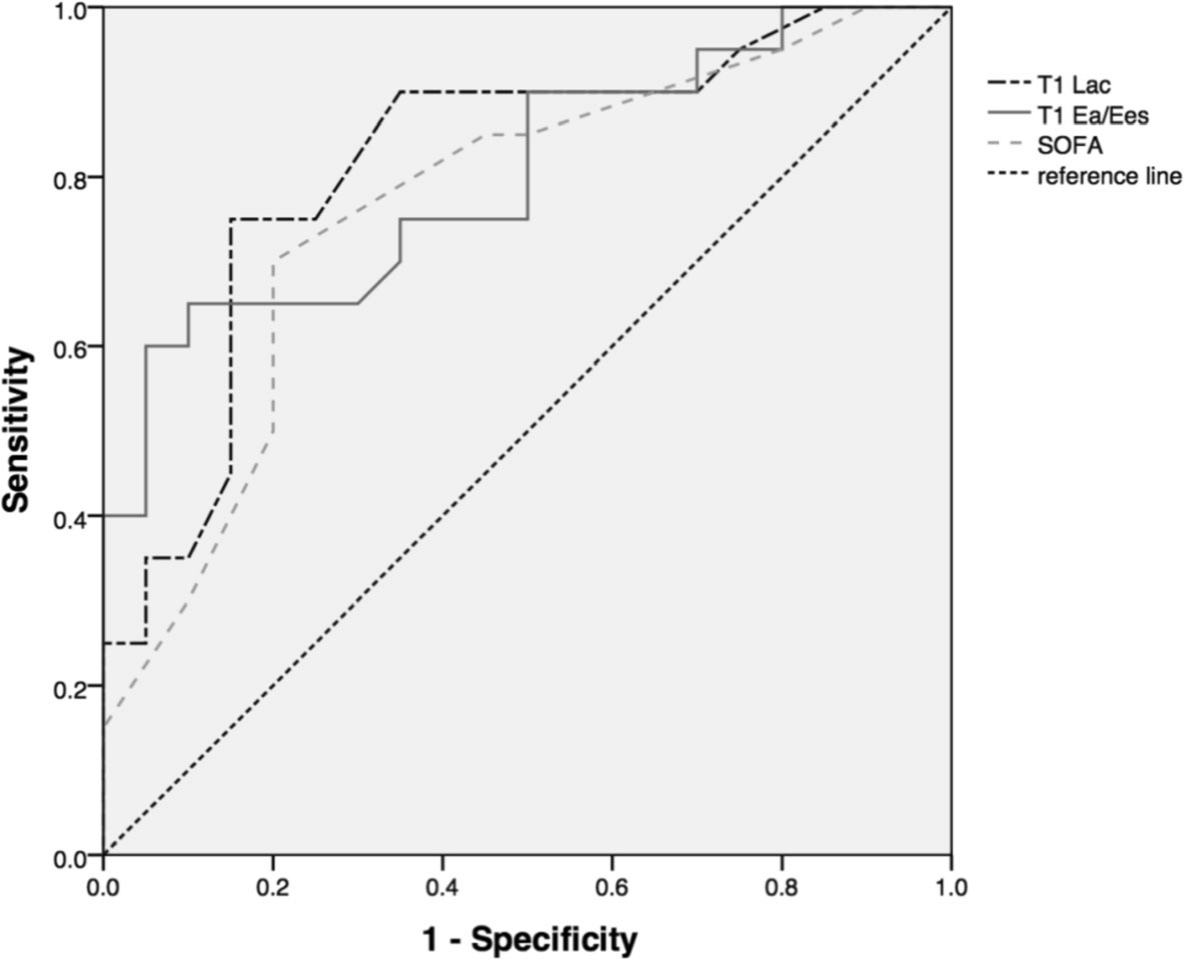
The ROC curves of lactate_T1_, Ea/Ees_,T1_ and SOFA for prolonged MV in post-cardiac surgery patients

ROC curves were drawn to compare the predictive values of three different variables for PMV (Fig. 4). The AUC demonstrated that the predictive values of SOFA, Ea/Ees_,T1_ and lactate_T1_ were 0.766 (95% CI:0.680–0.952), 0.801 (95% CI:0.664–0.938), 0.816 (95% CI:0.680–0.952), respectively (Table 4). The cutoff values for SOFA (1st day in the ICU), Ea/Ees_,T1_ and lactate_T1_ were 12.5 (sensitivity: 70%; specificity: 80%), 5.12 (sensitivity: 65%; specificity: 90%) and 1.60 (sensitivity: 75%; specificity: 85%), respectively, based on the maximum Youden index.

**Table 4:** The results of ROC analysis for Lac_T1_, Ea/Ees_,T1_ and SOFA

Variables	ROC area	95%CI	Cutoff value	Sensitivity	Specificity
Lac (mmol/L)	0.816*	0.680–0.952	1.60	75%	85%
Ea/Ees	0.801*	0.664–0.938	5.12	65%	90%
SOFA	0.766*	0.616–0.916	12.5	70%	80%

*Lac* lactate, *Ea/Ees* ventricular arterial coupling, *SOFA* sequential organ failure assessment

^*^P < 0.05

## Discussion

The main findings of this study are as follows: (a) VAC is associated with the duration of MV, and a worse VAC indicates a longer duration of MV; (b) VAC is an independent risk factor for PMV. A cut-off value of VAC > 5.12 predicts PMV with 65% sensitivity and 90% specificity.

Prolonged MV is associated with many complications [17, 18], such as ventilator-associated pneumonia, ventilator-induced diaphragm dysfunction, increased use of medical resources, etc. [3, 17]. Clinicians aim to reduce the duration of MV as quickly as possible. However, for severe post-cardiac surgery patients, early weaning is much more difficult. Low CO or impaired contractility has always been considered a risk factor for PMV [8] . However, patients who have a normal CO before weaning can still be difficult to wean [9, 19]. Hence, CO might not be adequate for predicting PMV. We also found that in severe post-cardiac surgery patients, the cardiac index was not associated with the duration of MV and could not predict PMV.

### The role of left VAC in prolonged MV

When weaning begins, the cardiac preload and afterload of the LV increase dramatically, which requires the LV to increase its SW. If a patient’s heart has a greater SW reserve before weaning, which indicates better VAC, it will be much easier to achieve adequate SW and wean the patient earlier. In several previous studies, SW before weaning was considered a predictive factor for PMV [7]. Actually, a heart can maintain adequate SW by sacrificing its reserve. Under this circumstance, CO or SV may be not altered, but the VAC will be changed. Therefore, it is the SW reserve and not the absolute SW that is important for early weaning. Our study confirmed this with the finding of no correlations between CI or SVI and the duration of MV in severe post-cardiac surgery patients.

Several studies [20, 21] have recommended VAC reserve as a significant tool for assessing cardiovascular function and prognosis. Tonino [20] et al. found that the change in VAC during stress echocardiography can be used as to predict adverse events in patients with negative stress echocardiography. In their study, they defined the change as the VAC reserve (VAC reserve = VAC_before_-VAC_after_), which can reflect the cardiac compensatory function capability. Kensuke [21] et al. also proved that a good VAC reserve was an important determinant of cardiovascular outcome for patients with dilated cardiomyopathy. In the present study, we found that an increased Ea/Ees_,T1_ (OR 6.840; 95% CI 1.031–45.359, *P* < 0.05) was a risk factor for prolonged MV. Hence, the assessment and optimization of the left VAC might be helpful during ventilator weaning in severe post-cardiac surgery patients. Further studies are required to validate VAC-directed weaning from ventilation.

### Optimizing VAC during weaning

Vasodilators and inotropes are always used in severe post-cardiac surgery patients to assist in weaning from MV, which also optimizes VAC. Vasodilators, which decrease Ea by reducing the afterload, and inotropes, which augment Ees by strengthening contractility [22], are two important therapies that can improve VAC, which is crucial for ensuring a short duration of MV. Several studies have shown that vasodilators and inotropes can facilitate the weaning process [9, 23]. Routsi [9] et al. used a nitroglycerin infusion to control systolic blood pressure, and it greatly improved the weaning process of difficult-to-wean patients. Despite concerns about inotrope use, the researchers verified that levosimendan and dobutamine can facilitate weaning in difficult-to-wean COPD patients [24]. Moreover, fluid management also be favorable for VAC. Teboul [5] et al. suggested fluid removal as a reasonable option to help difficult-to-wean patients. Removing excessive fluid can reduce left ventricular end-diastolic pressure [25], which can increase coronary perfusion pressure and ameliorate myocardial perfusion [26].

Several limitations should be acknowledged. First, leaving V_0_ out of the calculation formula and using GEDV/4 as LVEDV might be an arbitrary decision. However, we had several reasons for making this decision: (a) we considered the heart as a whole, because PMV is not only a problem of poor left ventricular function but also a thing of right ventricle [27]. It might be more proper to combine right and left ventricle together, so we used GEDV/4, which can take both cardiac chambers into consideration, to address this problem. (b) the value of GEDV as a reflection of the preload of the LV has been confirmed in many studies [28], and (c) GEF, calculated by 4SV/GEDV, has a good correlation with LVEF, calculated by SV/LVEDV [29]. Most importantly, in 2015, our department had done a research about sepsis and this “revised VAC” previously, and confirmed its significance in managing sepsis. Further studies are required to validate these choices; however, to some extent, they were reasonable. Second, this was a single-center study with a small sample size. Further investigations with large sample sizes need to be completed to confirm our results. Third, VAC is influenced by many factors: gender, age, concomitant diseases, etiologies, etc. [30–32]. We analyzed these possible variables and found no differences between the two groups. In addition, there were no differences in VAC at admission. We believe that the significance of the differences in VAC was not caused by these irrelevant factors. Fourth, in this study, we aimed to determine what we should do before weaning and tried to recognize patients at risk for prolonged MV patients early in their stay; consequently, we chose the first 48 h as our study period [33, 34]. Fifth, existing research leaves no doubt that VAC is a measure of cardiac work efficiency. Considering our thorough descriptions of VAC and its role in prolonged MV, there is adequate support for our views and conclusions. Further studies must be performed to confirm our perspectives in practice.

## Conclusion

Compared to CO, VAC is better in predicting PMV in severe post-cardiac surgery patients, and it’s also an independent risk factor for PMV, which means poorer VAC indicates higher possibility of PMV. The assessment and optimization of left VAC might be helpful when weaning severe post-cardiac surgery patients from MV.

## Abbreviations

CABG: Coronary artery bypass grafting
CAC: Coronary atherosclerotic cardiopathy
CHF: Chronic heart failure
CI: Cardiac output index
CKD: Chronic kidney disease
CO: Cardiac output
COPD: Chronic obstructive pulmonary disease
CVP: Central venous pressure
Ea: Arterial end-systolic elastance
Ea/Ees: Ventricular arterial coupling
ECC: Extracorporeal circulation
Ees: Left ventricular end-systolic elastance
GEDV: Global end-diastolic volume
GEDVI: Global end-diastolic volume index
HR: Heart rate
Lac: Lactate
LV: Left ventricle
MV: Mechanical ventilation
MVO_2_: Myocardial oxygen consumption
P/F: PaO2/FiO2
PMV: Prolonged mechanical ventilation
PVA: Pressure–volume area
ROC: Receiver operating characteristic
SBP: Systolic blood pressure
SBT: Spontaneous breathing test
SOFA: Sequential organ failure assessment
SV: Stroke volume
SVI: Stroke volume index
SVRI: Systemic vascular resistance index
SW: Stroke work
VAC: Ventricular-arterial coupling
